# Editorial: Nucleosides, nucleotides and nucleic acids: chemistry and biology

**DOI:** 10.3389/fchem.2024.1401510

**Published:** 2024-04-10

**Authors:** Leandro Soter de Mariz e Miranda, Raoni Schroeder Borges Gonçalves, Jacques Uziel, Satoshi Obika, Nadege Lubin-Germain

**Affiliations:** ^1^ Biocatalysis and Organic Synthesis Group, Universidade Federal do Rio de Janeiro, Centro de Tecnologia, Rio de Janeiro, Brazil; ^2^ CY Cergy-Paris Université, BioCIS, CNRS, Cergy, France; ^3^ Université Paris-Saclay, CNRS, BioCIS, Châtenay-Malabry, France; ^4^ Graduate School of Pharmaceutical Sciences, Osaka University, Suita, Japan

**Keywords:** nucleosides, nucleotides, nucleic acids, metal-dependant DNA methylation level, therapeutic agents, enzymatic synthesis, vorbruggen conditions, phosphate mimics

Nucleosides and nucleotides constitute the elementary building blocks of nucleic acids, fundamental molecular components of life playing an essential role in heredity by transmitting and storing genetic information (Minchin and Lodge, 2019). Here we bring together the contributions on this Research Topic and will address the problem of synthesis, epigenetics and the therapeutic approach (Liu et al.; Sabat et al.; Berdis; Naciuk et al.; Sergeeva et al.).

DNA expression depends on nucleotides post replication chemical modifications. One of them is the DNA methylation occurring exclusively at C-5 in the pyrimidine ring of cytosine as an epigenetic marker in CpG dinucleotides promoters. The methylation level is directly connected to the promotion or dysfunction of biological processes such as carcinogenesis. The question of factors disrupting the methylation equilibrium is of a great interest and Liu et al. explored the role of metals on the DNA methylation level. The authors confirmed the effects of metal ions on DNA methylation in a sequence-specific manner using fluorescent *in situ* hybridization (FISH) methodology.

Nucleic acids are also involved in numerous cellular processes such as cell signaling (ATP as an energy source and cAMP as a second messenger transmitting information inside the cell), protein translation by transfer RNAs which delivers the correct amino acid or duplication processes (DNA replication or transcription into messenger RNA) using the constituent building blocks.

The synthesis of nucleic acids by molecular biology techniques such as Polymerase Chain Reaction (PCR) enables access to macromolecular diversity with good fidelity and sufficient quantity. The best example is the discovery and production of *m*-RNA vaccines such as the one against COVID-19. Several vaccines are already in clinical trials for infectious diseases (influenza, Zika virus, Nipah virus, respiratory syncytial virus), and for genetic disorders and cancers (Khan et al., 2023). Moreover, DNA being a system coded by 4 nucleobases, it has been considered in recent years as a valuable medium for storing information to meet the energy costs of current servers. DNA has a sufficiently stable chemical structure since it has been possible to sequence the mammoth genetic code, and it can be produced in the form of a very long chain by PCR (Doricchi et al., 2022).

In addition to molecular biology techniques, nucleic acids can be obtained by synthesis using the couplings of phosphoramidite nucleotide derivatives. This technique employs chemical reactions to sequentially add nucleotides to a solid matrix, following the specified desired sequence for antisense oligonucleotide ASO (Duffy et al., 2020). Even if both conditions are efficient, sustainability and scalability require new improvements as suggested by Hollenstein *et al.* who considered an enzymatic approach using a template-independent polymerase such as the terminal deoxynucleotidyl transferase (TdT) or Poly(U) polymerases (Sabat et al.) with C3′-protected (locked) nucleotides. In addition, Sergeeva et al. were interested in alternative phosphate mimics for RNA therapeutics. They proposed to advantageously replace phosphothiolate bonds, most often proposed for ASO gapmers, with *N*-alkane sulfonyl phosphoramidate groups such as mesyl (methanesulfonyl) or busyl (1-butanesulfonyl) phosphoramidates to improve biological properties, RNase H cleavage, cellular uptake *in vitro*, and intracellular localization.

Concerning nucleosides and nucleotides, they have been of particular interest for several decades following the HIV virus discovery. The therapeutic success of the nucleoside Zidovudine (azidothymidine) A therapeutic approach has led to the synthesis of numerous families of active and specific antiviral nucleosides. In addition, the development of broad-spectrum nucleosides opens up possibilities for combined therapy. Recently, the prodrug approach leading to the *in cellulo* delivery of nucleotides has led to significant advancements, as demonstrated in the treatment of hepatitis C with sofosbuvir. As another example, A. Berdis (Berdis) reports pre-clinical studies using nucleobase-modified nucleosides as potential therapeutic agents against diseases such as cancer. More generally the introduction of non-natural nucleotides is extensively studied to examine the contributions of shape/size, nucleobase hydrophobicity, and pi-electron interactions, to improve the strand complementarity in an antisense approach, or introduce modified nucleotide for synthetic biological uses.

Access to nucleosides and nucleotides analogues remains a challenge, as their therapeutic interest is so important. This requires efficient and stereochemically controlled synthetic methodologies. Naciuk et al. revisit the Vorbruggen coupling reaction for weakly reactive nucleobases, in the presence of Lewis acids. The authors then describe the synthesis of 7-deaza-2′-methyladenosine (7DMA) employing Vorbrüggen conditions, as well as preliminary results of efficacy against an emerging flavivirus *in vitro*.

As evident, the chemistry and biology of these compounds, from the elementary building blocks to biopolymers, are rich both synthetically and in their biomedical applications. If complexity is demonstrated by sequence (DNA *versus* RNA), supramolecular aspects (messenger, transfer, cyclic … ) their conformational properties (single/double strand, quadruplexes … ) have to be considered as well. For all these reasons, progress associated with this field has been significant in recent years, and it seems important to take a specific interest.
